# Association between Residential Greenness and Incidence of Parkinson’s Disease: A Population-Based Cohort Study in South Korea

**DOI:** 10.3390/ijerph19063491

**Published:** 2022-03-15

**Authors:** Jiyun Jung, Jae Yoon Park, Woojae Myung, Jun-Young Lee, Hyunwoong Ko, Hyewon Lee

**Affiliations:** 1 Data Management and Statistics Institute, Dongguk University Ilsan Hospital, Goyang 10326, Korea; bestjudy21@gmail.com; 2 Research Center for Chronic Disease and Environmental Medicine, College of Medicine, Dongguk University, Goyang 10326, Korea; nephrojyp@gmail.com; 3 Department of Internal Medicine, Dongguk University Ilsan Hospital, Goyang 10326, Korea; 4 Department of Internal Medicine, College of Medicine, Dongguk University, Goyang 10326, Korea; 5 Department of Neuropsychiatry, Seoul National University Bundang Hospital, Bundang-gu, Seongnam 13620, Korea; smbhealer@gmail.com; 6 Department of Psychiatry, College of Medicine, Seoul National University, Seoul 08826, Korea; 7 Department of Psychiatry, SMG-SNU Boramae Medical Center, College of Medicine, Seoul National University, Seoul 08826, Korea; benji@snu.ac.kr; 8 Interdisciplinary Program in Cognitive Science, Seoul National University, Seoul 08826, Korea; powerzines@gmail.com; 9 Department of Health Administration and Management, College of Medical Sciences, Soonchunhyang University, Asan 31538, Korea; 10 Department of Software Convergence, Soonchunhyang University Graduate School, Asan 31538, Korea

**Keywords:** Parkinson’s disease, residential greenness, long-term exposure, cohort study

## Abstract

It is widely known that exposure to residential greenness is beneficial for health. However, few studies have analyzed the association between greenery and Parkinson’s disease (PD). We selected 313,355 participants who matched the inclusion criteria from the National Health Insurance Service-National Sample Cohort, followed up from 2007 to 2015. Residential greenness, represented by the normalized difference vegetation index (NDVI), was obtained from satellite measurements. We estimated hazard ratios of PD associated with a 0.1-unit increase in long-term greenness exposure at the district level for the previous 1 year of each year until a censoring/event occurred, using time-varying Cox proportional hazard models, adjusted for individual- and area-level characteristics. During the 2,745,389 person-years of follow-up, 2621(0.8%) participants developed PD. Exposure to higher levels of residential greenness was found to be associated with a decreased risk of PD incidence (21% per 0.1-unit increase, 95% confidence interval (CI): 0.74–0.84). In subgroup analyses, stronger protective effects were observed in participants aged over 50 years, females, overweight/obese participants, non-urban residents, non-smokers, alcoholics, and those with comorbidities. Long-term exposure to greenness was beneficial to incident PD, and our findings could aid in the development of public-health strategies.

## 1. Introduction

Parkinson’s disease (PD) is the second most prevalent neurodegenerative disease after Alzheimer’s disease (AD), posing a great socio-economic burden to our aging society [1]. Globally, 6.1 million individuals had PD in 2016, which was much higher than 2.5 million PD cases in 1990, and disability-adjusted life-years and deaths due to PD were 3.2 million and 211,000 in 2016, respectively [2]. Given the rapid increase in the aging population worldwide, the global burden of PD is expected to increase drastically in the coming decades [2,3]. Moreover, many motor and non-motor processes are affected by PD, including tremor, bradykinesia, depression, and cognitive dysfunction [4,5], all of which contribute to patients’ poor quality of life [6] and even suicide [7]. Despite its severity, currently there is no cure for PD, and establishing basic preventative treatment measures can reduce the disease’s growing burden.

Considering that 90% of PD cases have no distinct genetic cause [8], many previous studies have identified behavioral or environmental protective factors for PD, including smoking, caffeine consumption, use of ibuprofen, and physical activity [9], though only physical activity could be a reasonable intervention. Recently, residential greenness has been widely examined as an emerging environmental factor that reduces various health comorbidities, including neurological diseases [10,11,12,13]. Good residential greenness has been associated with reduced prevalence of depression [13], slower cognitive decline [14], increased cortical thickness [15], reduced mortality from neurological diseases [11], and reduced risk of AD and non-AD dementia [12]. This recent evidence supports the hypothesis that residential greenness could be a neuroprotective factor for PD. To the best of our knowledge, there have been only two epidemiological studies assessing the association between residential greenness and the risk of PD: One study reported a significant protective effect of residential greenness for incident PD using 47,516 cohort participants in Ningbo, China [16], and the other study reported a marginal protective effect based on 634,432 participants in Metro Vancouver, Canada [17].

To contribute to the limited evidence on the protective role of residential greenness in PD, we conducted a national-level cohort study to investigate the association between residential greenness and the incidence of PD using a nationwide population-based cohort of South Korean citizens.

## 2. Materials and Methods

### 2.1. Data Source and Study Population

Data for this nationwide population-based cohort study were obtained from the National Health Insurance Service-National Sample Cohort (NHIS-NSC, ver. 2.0). The NHIS is a universal insurance provider that was launched in 2000 as a single-payer health insurance system for all citizens of South Korea [18]. The NHIS established the National Health Insurance Database (NHID), which contains medical records of all citizens, including sociodemographic information (sex, age, insurance premium, and residential addresses), healthcare utilization (outpatient visits or admission), medication prescriptions, and national health examination results. From the 2006 NHID, the NHIS created the NHIS-NSC of one million individuals, who were randomly and proportionally sampled based on sex, age, residential area, and insurance premium from the total 48 million population of South Korea. The NHIS-NSC includes individuals’ information for 2002–2015 merged retrospectively and prospectively.

We selected 329,491 participants who had undergone a national health examination at least once between 2002 and 2006 from one million individuals in the NHIS-NSC because data on important PD-related factors—including smoking status, physical activity, alcohol consumption, and body mass index—were obtained from the national health examination [9]. In addition, we excluded participants who had died before 2007 (*n* = 0) and were diagnosed with PD before 2007 (*n* = 680), those with incomplete address information (*n* = 1579), and those with incomplete information for any covariates (*n* = 13,877). Finally, 313,355 participants were included in this cohort study (Figure S1).

### 2.2. Primary Outcome

The primary outcome of this study was the incidence of PD. PD was identified based on the International Classification of Diseases, 10th Revision (ICD-10) code G20, and medication prescriptions. The incidence of PD was defined as the first healthcare utilization (admission or outpatient visit) due to PD as a primary or secondary diagnosis for 2007–2015, simultaneously accompanied by the prescription of anti-PD medications such as carbidopa and levodopa.

### 2.3. Residential Greenness

Exposure to residential greenness was represented by the satellite-derived NDVI, which has been widely used to estimate the effects of vegetation on mortality [19] and morbidity [20]. NDVI obtained by the Moderate Resolution Imaging Spectroradiometer (MODIS) from aboard Terra satellites has been produced at 16-day intervals at a 250 m spatial resolution since 2000 [21]. NDVI was calculated as the land surface reflectance using near-infrared radiation (NIR) and visible red, because green vegetation absorbs red wavelengths but reflects NIR compared to non-green vegetation [22]. Therefore, given the mathematical formula (NIR − red)/(NIR + red), negative values close to −1 indicate soil and area without vegetation, while positive values close to 1 indicate a high density of green vegetation. We determined the daily NDVI for 16 days between 2000 and 2017 by 282 spatial units named Si-Gun-Gu, which is the practical administrative district unit in Korea, and assigned annual average individual exposure of NDVI from the previous 1–5 years at baseline to censoring date by considering the annually updated participants’ residence. Exposure to greenness within the buffer area could not be measured because of a lack of data, although a previous study considered exposure to greenness in buffer areas having radii of 300 m and 1000 m around a residential address [16].

### 2.4. Statistical Analyses

**Figure 1:**
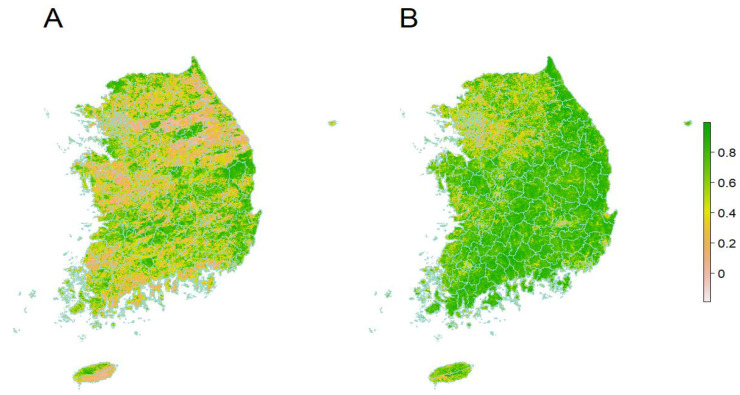
Residential greenness on July 2006 (**A**) and 2014 (**B**).

Time-varying Cox proportional hazard regression was conducted to assess the hazard ratios (HRs) and 95% confidence intervals (CIs) of PD incidence associated with annually updated exposure to NDVI from the previous 1 year at baseline. The time-varying model reflects the variability in exposure that changes over time. Survival time was measured in years from baseline (1 January 2007) until PD incidence date, mortality date, or end of follow-up (31 December 2015). Figure 1 shows the distribution of residential greenness on July 2006 (A) and 2014 (B). Thus, the time-varying model was suitable to reflect the distribution of green areas that change over time. We applied the NDVI as a continuous and categorical variable. Based on the quartile of previous 1-year NDVI exposure at baseline, we estimated the HRs of each quartile compared to the lowest quartile as reference and tested the linear trend using the ordinal rank for each quartile. For continuous NDVI, the HRs by a 0.1 increase in NDVI were estimated.

We explored the effects of continuous and categorized NDVI on PD incidence in several models. Model 1 is a crude model adjusted for age and sex. Model 2 was additionally adjusted for individual characteristics such as residential province, household income (three levels: low, medium, and high), insurance type (four levels: self-insured, dependent on self-insured, employer-insured, and dependent on employer-insured citizens), alcohol consumption (three levels: rarely, sometimes, and frequently), smoking status (three levels: never, former, and current), exercise (three levels: rarely, sometimes, and frequently), body mass index (BMI), fasting glucose, total cholesterol, frequency of healthcare utilization, history of traumatic brain injury and mental disorders, Charlson comorbidity index (CCI), and medication history (ibuprofen, antipsychotics, and hormone). Model 3 is a fully adjusted model further adjusted by regional characteristics such as percentage of highly educated (high school graduation or over) people in the 2000 census, percentage of elderly (≥65 years) people in the 2000 census, gross regional domestic product in 2005, type of area (three levels: urban, suburban, rural), population density in 2008, and mean concentration of particles <10 μm (PM_10_). Personal exposure to the annual average of PM_10_ was estimated in the district (Si-Gun-Gu) unit by inverse distance weighting (IDW), which considers the square of the inverse distance between monitoring stations as weight [23]. IDW is one of the most widely used spatial interpolation methods that predicts the unmeasured values using the nearest points to the location, and is suitable because monitoring stations are non-equally distributed nationwide.

To investigate more affected subgroups in the association, we conducted stratified analyses with exposure to greenness as continuous variable by key confounding factors [1] including sex, age (<50, ≥50 years), BMI (<25, ≥25 kg/m^2^), residential province (seven metropolitan cities and nine provinces), smoking status (never, ever), alcohol consumption (rarely, sometimes/frequently), and comorbid states (CCI 0, 1–17). In addition, several sensitivity analyses were conducted to test the robustness of the long-term effects of NDVI in a continuous term on PD in the main model. First, we assessed the effects of different exposure windows of NDVI from the previous 2 to 5 years. Second, we applied conventional Cox proportional hazard regression models with baseline NDVI exposure from the previous 1 year to 5 years. Third, we restricted the study population to non-movers during the study period. Finally, we adjusted for nitrogen dioxide (NO_2_), sulfur dioxide (SO_2_), and carbon monoxide (CO), instead of PM_10_, to examine possible confounding effects of various air pollutants.

All analyses were conducted using SAS Enterprise Guide version 7.2 (SAS Institute Inc., Cary, NC, USA) and R Studio version 1.0.136 (packages survival and survminer).

## 3. Results

Among 313,355 participants over 2,745,389 person-years of follow-up, we observed 2621 incident PD patients between 2007 and 2015 (Table 1). The mean (standard deviation; SD) age of the total population was 48.9 (14.2) years, and 35.6% of participants were in the 35–49 age group. More than half (54.1%) were male and less than half (48.5%) were employer-insured citizens, most of them rarely consumed alcohol (71.5%), never smoked (66.0%), and rarely exercised (81.5%). Compared to the study participants in the lowest quartile of greenness, those in the highest quartile of greenness were older, more likely to consume alcohol, exercise frequently, and had more incident PD events and comorbid diseases, and lived in provinces with distinct regional characteristics (higher proportion of the elderly, lower proportion of highly educated people, low gross regional domestic product, and population density). Table 2 presents the summary statistics of the NDVI exposure and air pollution concentrations of the study participants for the first study year, 2007. The mean (SD) of NDVI exposure for the previous 1 year was 0.41 (0.11), and those for the previous 2–5 years were similar, ranging from 0.40 (0.11) to 0.42 (0.12). The 1-year average concentrations of PM_10_, SO_2_, NO_2_, and CO were 59.25 μg/m^3^, 5.76 ppb, 27.06 ppb, and 0.82 ppm, respectively.

Table 3 shows the estimated HRs (95% CIs) of PD incidence associated with exposure to continuous and categorized NDVI in the previous 1 year. In Model 3, where individual- and area-level covariates were fully adjusted, a 0.1 unit increase in NDVI was significantly associated with a 21% decrease in the risk of incident PD (95% CI, 0.74–0.84). In the categorized NDVI analysis, exposure to the highest quartile of NDVI compared to the lowest quartile was associated with a 36% decreased risk of incident PD (95% CI, 0.52–0.77), showing a linear trend (*p* for trend = 0.01). In Model 1 and Model 2, where individual and/or area-level covariates were not adjusted, the association between NDVI and PD was negative but not significant.

In stratified analyses, we identified more affected subgroups per a 0.1-unit increase in NDVI (Table 4) as follows: females (HR: 0.76, 95% CI: 0.69–0.83), participants with high BMI (≥25 kg/m^2^) (HR: 0.76, 95% CI: 0.68–0.85), those living in provinces (HR: 0.72, 95% CI: 0.66–0.79), never smokers (HR: 0.77, 95% CI: 0.71–0.83), sometimes/frequent drinkers (HR: 0.83, 95% CI: 0.70–0.98), and those with comorbidities showed stronger effect estimates (HR: 0.77, 95% CI: 0.72–0.83) than males (0.83 [0.75–0.91]), those with low BMI (HR: 0.80, 95% CI: 0.74–0.87), those living in metropolitan cities (HR: 0.90, 95% CI: 0.81–0.99), ever smokers (HR: 0.87, 95% CI: 0.75–1.00), rare drinkers (HR: 0.94, 95% CI: 0.87–1.00), and those without comorbidities (HR: 0.84, 95% CI: 0.73–0.96). The effect estimates were similar between younger (HR: 0.78, 95% CI: 0.61–1.00) and older participants (HR: 0.79, 95% CI: 0.74–0.84), but the association in younger participants was not significant.

Our main findings were that the significant inverse associations between residential greenness and incident PD were robust to several sensitivity analyses. The analyses with longer exposure windows of NDVI yielded consistent results with the main findings (Table S1), while the conventional time-constant Cox analysis (satisfied proportional hazards assumption; *p* > 0.05) resulted in decreased effect estimates (HR 0.92–0.93) (Table S2). The analysis restricted to 173,661 non-movers showed similar results to the main results (Table S3). Finally, the analyses adjusting for SO_2_, NO_2_, and CO instead of PM_10_ also showed consistent results with the primary findings (Table S4).

## 4. Discussion

In this nationwide population-based cohort study, we investigated the association between long-term exposure to residential greenness and incident PD events after adjusting for demographic, lifestyle, physical, laboratory, and regional characteristics, as well as exposure to ambient air pollution. We found that exposure to higher levels of residential greenness significantly decreased the risk of PD. Stronger inverse associations were observed in participants aged over 50 years, females, overweight/obese participants, non-urban residents, never-smokers, alcohol consumers, and those with comorbidities. Our primary findings were consistent with those of various sensitivity analyses

To the best of our knowledge, our study is the first national-level cohort study on residential greenness and incident PD based on 313,355 nationwide-representative cohort participants. To date, only two epidemiological studies have investigated the association between long-term greenness exposure and PD [16,17]. Using a prospective cohort of 47,516 participants aged over 18 years in Ningbo, China, between 2015 and 2018, a previous study found a significant protective effect of residential greenness on PD incidence, which corresponds to our findings [16]. The effect estimates from the previous study were also similar to our findings in both the linear NDVI (HR per IQR [0.25] increment: 0.80, 95% CI: 0.65–0.98) and the categorized NDVI analyses (HR for Q4 [>0.51] vs. Q1 [≤0.26] of greenness within radii of 300 m around residence: 0.57, 95% CI: 0.37–0.85), although our effect estimates were slightly higher. A possible reason for the slight difference may be the different spatial resolution used to define residential greenness; our study used district-level exposure based on a 250 m buffer, whereas the previous study used a 300 m buffer around residences. Additionally, differences in several factors—including vegetation status (median [Q1–Q3] of NDVI: 0.41 [0.34–0.50] vs. 0.38 [0.26–0.51]), outcome definition (ICD-10 code combined with medication use vs. ICD-10 code), study area, and study population—might also contribute to the slightly higher effect estimates in our study. Another cohort study of 634,432 participants aged 45–85 years in Metro Vancouver, Canada between 1999 and 2003 reported a marginal protective effect of greenness in 100 m radius buffer on PD incidence (HR per IQR [0.11] increment: 0.96, 95% CI: 0.92–1.01), which was consistent with our findings [17]. Due to restricted data availability, the 1999–2002 greenness average was assigned as the baseline (1999) exposure and a time-constant Cox model was utilized, which could have resulted in increased exposure measurement error. Moreover, the annual greenness exposure in the previous study ranged from −0.11 to 0.78, while that in our study ranged from 0.16 to 0.64.

Recent studies have studied the neurological effects of residential greenness and reported significant neuroprotective effects, including reduced depression [13], slower cognitive decline [14], increased cortical thickness [15], reduced mortality from neurological diseases [11], and reduced risk of AD and non-AD dementia [12]. Although this study provides evidence about the neurological effect of residential greenness based on PD incidence, there is still a dearth of information, requiring further studies to elucidate the effects of greenness on neurological disorders.

The biological pathways of residential greenness in neurological disorders, including PD, remains unclear; however, greenness exposure would be associated with a lower chance of developing PD. Greenness may encourage higher levels of moderate or vigorous physical activities [24], such as walking or jogging, which promotes neural growth factors, contributing to the survival of dopaminergic neurons [25]. In addition, green infrastructure could mitigate the impact of atmospheric particles, which causes neurotoxicity and neurodegeneration through oxidative stress by dry deposition onto the leaves of vegetation [26,27]. Given the limited evidence on the biological mechanisms, future studies focusing on the biological mechanisms of nervous system diseases associated with greenness exposure should be conducted.

Our findings of the stronger inverse associations in older participants (≥50 years), overweight/obese participants, alcoholics, and those with comorbidities can be explained by the susceptibility of these subgroups, implying that exposure to greenness could be more advantageous to sensitive subgroups. In the sex-specific analysis, we observed a stronger protective effect in females, which is consistent with the findings of a previous PD study in Ningbo, China [16]. Studies on other health outcomes besides PD found inconsistent results regarding the sex-specific effects of greenness; some studies reported a stronger effect in females [28,29,30], while several studies reported a stronger association among males [31,32]. The difference might be explained by different behavioral patterns in which males are more likely to be more active than females in green spaces [33], but this could not be verified in this study. Further studies on different behavioral patterns, as well as biological differences among males and females, could help to elucidate the effects of greenness on health, including PD.

The stronger protective effect of greenness in never smokers than ever smokers in our study is consistent with a recent study from Ningbo, China. [16]. The study found that the highest quartile of greenness exposure compared to the lowest quartile was significantly associated with a lower risk of incident PD in never smokers (HR, 0.53; 95% CI: 0.34–0.83) but not in ever smokers [16]. Similarly, a longitudinal cohort study on the association between residential greenness and mortality reported a stronger association in never smokers (HR: 1.09, 95% CI 1.04–1.14) than in smokers (HR: 1.03, 0.91–1.17) [34]. In contrast, smokers benefitted more from the effects of greenness than never smokers with decreased blood pressure [35] and the prevalence of chronic kidney disease [36]. Therefore, both smokers and never-smokers can benefit from exposure to residential greenness.

We found a noticeable protective effect of greenery on PD in provinces than in metropolitan cities. Aside from Parkinson’s disease, for which no previous research has been done, various studies have looked into the relationship between urbanity and cancer mortality [37], general health [38], hypertension [39], and cardiovascular mortality [40]. Research on cancer mortality [37] and general health [38] indicated a stronger effect of residential greenness in urban areas than in rural regions, whereas studies on hypertension [39] and cardiovascular mortality [40] found a stronger effect of residential greenness in rural areas. The possible urban–rural differences can be explained by several factors, including the amount of residential greenness [41], accessibility to green space [42], types of vegetation, and structure of green space [43], which need to be validated in further research.

Our study has several limitations. First, our exposure assessment based on district-level residential addresses may not reflect accurate individual exposure and lead to exposure misclassification, despite the fact that we obtained high spatial resolution greenness measurements from satellites. However, district-level residential greenness assessments may be more accurate in capturing participants’ travel from home to work or other activities. Moreover, the NDVI captured by the satellite could not indicate vegetation type [44] or green structure [45] which could be possible confounders in the association between greenness and incident PD; however, NDVI could be an indicator of objectively measured vegetation density. Second, other potential confounders—such as occupation, education level, genetic factors, and caffeine intake—could not be adjusted due to limited data availability, although we extensively adjusted various sociodemographic and laboratory data as well as area-level characteristics. Lastly, the outcome ascertainment using diagnostic codes might have caused outcome misclassification; however, the possibility of misclassification is low because, in South Korea, the government pays 90% of medical expenses for PD, and PD patients must obtain confirmatory diagnosis of PD using imaging examinations to be eligible for these benefits.

Despite several limitations, our research has a number of advantages. First, we conducted a national-level cohort analysis based on the government’s largest and most trustworthy national claims database, which contains actual healthcare utilization of Korean citizens. Second, important individual-level covariates were addressed in this study, such as medication history, lifestyle (smoking/drinking/exercise), laboratory findings, and concomitant disorders. Third, we employed spatial interpolation of air pollution to overcome the limitations of monitoring stations concentrated in urban areas to adjust for the primary confounding factor, long-term air pollution exposure. Finally, the results of various stratified studies by important features could aid in the identification of more benefited subpopulations and the development of effective health policies.

## 5. Conclusions

With no clear treatment for PD, it is important to identify the factors which protect against developing PD. Our study provides scientific evidence that living in higher levels of residential greenness could be beneficial for lowering the risk of PD incidence. With the rapid urbanization and rapid aging of population worldwide, our findings should be useful for policy makers and city planners to establish appropriate public health policies and interventions.

## Figures and Tables

**Table 1:** Baseline characteristics of 313,355 participants according to quartiles of normalized difference vegetation index.

Characteristics	Total (*n* = 313,355)	NDVI			
		Q1 (*n* = 78,959) [0.16–0.34]	Q2 (*n* = 78,025) [0.34–0.41]	Q3 (*n* = 79,503) [0.41–0.50]	Q4 (*n* = 76,868) [0.50–0.64]
PD Incidence, *n* (%)	2621 (0.8)	464 (0.5)	540 (0.6)	733 (0.9)	884 (1.1)
Age, mean (SD)	48.9 (14.2)	47.2 (13.5)	47.6 (13.6)	49.0 (14.3)	52.0 (14.8)
Age					
19–34	58,340 (18.1)	17,065 (21.6)	15,579 (20.0)	14,549 (18.3)	11,147 (14.5)
35–49	111,709 (35.6)	29,286 (37.1)	29,665 (38.0)	28,893 (36.3)	23,865 (31.0)
49–64	91,619 (29.2)	23,017 (29.2)	22,382 (28.7)	22,526 (28.3)	23,694 (30.8)
65–87	51,687 (16.5)	9591 (12.1)	10,399 (13.3)	13,535 (17.0)	18,162 (23.6)
Sex, *n* (%)					
Male	169, 561 (54.1)	42,896 (54.3)	42,868 (54.9)	43,421 (54.6)	40,376 (52.5)
Female	143,794 (45.9)	36,063 (45.7)	35,157 (45.1)	36,082 (45.4)	36,492 (47.5)
Income, *n* (%)					
Low (1st–3rd decile)	61,928 (19.8)	14,723 (18.6)	15,316 (19.6)	16,137 (20.3)	15,752 (20.5)
Medium (4–7th decile)	123,260 (39.3)	30,804 (39.0)	30,916 (39.6)	30,928 (38.9)	30,612 (39.8)
High (8–10th decile)	128,167 (40.9)	33,432 (42.3)	31,793 (40.7)	32,438 (40.8)	30,504 (39.7)
Insurance type, *n* (%)					
Self-insured citizens	50,702 (16.2)	12,051 (15.3)	11,953 (15.3)	12,910 (16.2)	13,788 (17.9)
Dependent of self-insured citizens	33,946 (10.8)	8109 (10.3)	7846 (10.1)	8452 (10.6)	9539 (12.4)
Employer-insured citizens	151,821 (48.5)	42,196 (53.4)	40,703 (52.2)	38,287 (48.2)	30,635 (39.9)
Dependent of employer-insured citizens	76,886 (24.5)	16,603 (21.0)	17,523 (22.5)	19,854 (25.0)	22,906 (29.8)
Alcohol consumption, *n* (%)					
Rarely	224,131 (71.5)	55,438 (70.2)	55,365 (71.0)	57,605 (72.5)	55,723 (72.5)
Sometimes	79,834 (25.5)	21,626 (27.4)	20,617 (26.4)	19,520 (24.6)	18,071 (23.5)
Frequently	9390 (3.0)	1895 (2.4)	2043 (2.6)	2378 (3.0)	3074 (4.0)
Smoking status, *n* (%)					
Never-smoker	206,828 (66.0)	51,400 (65.1)	50,962 (65.3)	53,151 (66.9)	51,315 (66.8)
Ex-smoker	27,413 (8.8)	7103 (9.0)	6809 (8.7)	6661 (8.4)	6840 (8.9)
Current smoker	79,114 (25.3)	20,456 (25.9)	20,254 (26.0)	19,691 (24.8)	18,713 (24.3)
Exercise, *n* (%)					
Rarely	255,510 (81.5)	63,961 (81.0)	63,045 (80.8)	65,409 (82.3)	63,095 (82.1)
Sometimes	40,166 (12.8)	11,080 (14.0)	10,797 (13.8)	9525 (12.0)	8764 (11.4)
Frequently	17,679 (5.6)	3918 (5.0)	4183 (5.4)	4569 (5.7)	5009 (6.5)
Health examination findings, mean (SD),					
Body mass index (kg/m^2^)	23.6 (3.2)	23.5 (3.2)	23.6 (3.2)	23.6 (3.3)	23.7 (3.2)
Fasting glucose (mg/dL)	95.4 (26.4)	94.4 (24.2)	95.3 (26.3)	95.5 (26.8)	96.6 (28.2)
Total cholesterol (mg/dL)	193.7 (41.5)	194.0 (43.6)	194.0 (41.2)	193.0 (38.8)	194.0 (42.3)
Healthcare utilization, *n* (%)					
Never	3776 (1.2)	1237 (1.6)	1023 (1.3)	820 (1.0)	696 (0.9)
Q1	70,947 (22.6)	20,487 (25.9)	18,759 (24.0)	16,880 (21.2)	14,821 (19.3)
Q2	80,002 (25.5)	20,961 (26.5)	20,794 (26.7)	20,118 (25.3)	18,129 (23.6)
Q3	80,134 (25.6)	19,861 (25.2)	19,798 (25.4)	20,673 (26.0)	19,802 (25.8)
Q4	78,496 (25.1)	16,413 (20.8)	17,651 (22.6)	21,012 (26.4)	23,420 (30.5)
Comorbidity, *n* (%)					
Traumatic brain injury	5056 (1.6)	1128 (1.4)	1138 (1.5)	1346 (1.7)	1444 (1.9)
Mental disorders	62,925 (20.1)	13,264 (16.8)	14,460 (18.5)	17,020 (21.4)	18,181 (23.7)
Charlson comorbidity index					
0	152,347 (48.6)	41,383 (52.4)	38,759 (49.7)	37,773 (47.5)	34,432 (44.8)
≥1	161,008 (51.4)	37,576 (47.6)	39,266 (50.3)	41,730 (52.5)	42,436 (55.2)
Medication history, n (%)					
Ibuprofen	96,986 (31.0)	23,538 (29.8)	24,422 (31.3)	26,630 (33.5)	22,396 (29.1)
Antipsychotics	5548 (1.8)	1210 (1.5)	1237 (1.6)	1867 (2.3)	1234 (1.6)
Hormone	11,288 (3.6)	2850 (3.6)	2773 (3.6)	2971 (3.7)	2694 (3.5)
Area characteristics ^a^, mean (SD)					
Residence, *n* (%)					
Metropolitan	141,408 (45.1)	65,658 (83.2)	37,736 (48.4)	26,834 (33.8)	11,180 (14.5)
Provinces	171,947 (54.9)	13,301 (16.8)	40,289 (51.6)	52,669 (66.2)	65,688 (85.5)
Percentage of highly educated people	50.6 (14.8)	56.4 (4.5)	53.8 (5.1)	50.2 (24.9)	41.8 (9.1)
Percentage of elderly people	7.4 (4.3)	5.2 (1.1)	5.6 (1.3)	7.9 (4.4)	11.1 (5.5)
Gross regional domestic product (won)	6,633,441 (4,536,448)	9,083,495 (3,342,065)	7,465,613 (3,951,047)	6,380,677 (5,248,281)	3,533,472 (3,397,134)
Population density (persons/km^2^)	6941 (7469)	15,900 (6521)	8214 (5618)	2753 (3108)	777 (1598)

^a^ For area-level characteristics, percentage of highly educated (high school graduates or more) people in 2000, percentage of elderly (aged ≥ 65 years) people in 2000, gross regional domestic product in 2005, and population density in 2008 were analyzed. Abbreviations: NDVI, normalized difference vegetation index; SD, standard deviation.

**Table 2:** Summary statistics of normalized difference vegetation index and air pollution concentrations for previous years of the first study year, 2007.

	Mean (SD)	Percentiles				
		0	25	50	75	100
NDVI						
Previous 1 year	0.41 (0.11)	0.16	0.34	0.41	0.50	0.64
Previous 2 years	0.42 (0.11)	0.18	0.34	0.42	0.51	0.64
Previous 3 years	0.42 (0.12)	0.16	0.33	0.42	0.51	0.66
Previous 4 years	0.40 (0.11)	0.16	0.32	0.41	0.49	0.61
Previous 5 years	0.42 (0.12)	0.16	0.34	0.43	0.51	0.65
Air pollutants						
PM_10_ (μg/m^3^)	59.25 (5.74)	40.30	55.19	59.41	63.55	74.29
SO_2_ (ppb)	5.76 (0.91)	3.82	5.18	5.79	6.17	10.04
NO_2_ (ppb)	27.06 (6.1)	11.47	22.11	25.46	32.69	38.91
CO (0.1 ppm)	0.82 (0.11)	0.49	0.78	0.85	0.89	1.18

Abbreviations: NDVI, normalized difference vegetation index; PM, particulate matter; SD, standard deviation.

**Table 3:** Association between exposure to normalized difference vegetation index and incidence of Parkinson’s disease in 1-year time-varying Cox hazards models.

	Person-Years	Cases	HR (95% CI)		
			Model 1 ^a^	Model 2 ^b^	Model 3 ^c^
Quantile					
Q1: 0.16–0.34	696,709	464	1 [Reference]	1 [Reference]	1 [Reference]
Q2: 0.34–0.41	687,668	540	1.15 (1.01–1.30)	1.12 (0.99–1.27)	0.96 (0.83–1.11)
Q3: 0.41–0.50	695,350	733	1.12 (1.00–1.26)	1.06 (0.94–1.19)	0.77 (0.65–0.91)
Q4: 0.50–0.64	665,662	884	1.03 (0.92–1.15)	0.97 (0.87–1.09)	0.64 (0.52–0.77)
*p* for trend			0.89	0.23	0.01
Linear per 0.1-unit increase	2,745,389	2621	0.98 (0.95–1.02)	0.97 (0.93–1.00)	0.79 (0.74–0.84)

^a^ Adjusted for age and sex. ^b^ Further adjusted for residential province, income, insurance type, alcohol consumption, smoking status, exercise, body mass index, fasting glucose, total cholesterol, frequency of healthcare utilization, history of traumatic brain injury and mental disorders, Charlson comorbidity index, and medication history (ibuprofen, antipsychotics, and hormone). ^c^ Further adjusted for district-level contextual information: percentage of highly educated (high school graduation or over) people (2000 census), percentage of elderly (≥65 years) people (2000 census), gross regional domestic product in 2005, type of area (urban, suburban, rural), population density in 2008, and mean concentration of PM_10_. Abbreviations: CI, confidence interval; HR, hazard ratio.

**Table 4:** Hazard ratios for association between long-term exposure to normalized difference vegetation index and Parkinson’s disease by age, BMI, residential province, and smoking per 0.1-unit increase.

	Person-Years	Cases	HR (95% CI)
Age			
<50	1,522,988	185	0.78 (0.61–1.00)
≥50	1,222,401	2436	0.79 (0.74–0.84)
Sex			
Male	1,479,836	1137	0.83 (0.75–0.91)
Female	1,265,553	1484	0.76 (0.69–0.83)
BMI			
<25 kg/m^2^	1,869,348	1651	0.80 (0.74–0.87)
≥25 kg/m^2^	876,041	970	0.76 (0.68–0.85)
Residence province			
Metropolitan	1,245,104	954	0.90 (0.81–0.99)
Provinces	1,500,285	1667	0.72 (0.66–0.79)
Smoking status			
Never	1,813,555	2075	0.77 (0.71–0.83)
Ever	931,834	546	0.87 (0.75–1.00)
Drinking			
Rarely	1,961,907	2187	0.94 (0.87–1.02)
Sometimes/frequently	783,482	434	0.83 (0.70–0.98)
CCI			
0	1,353,036	590	0.84 (0.73–0.96)
≥1	1,392,353	2031	0.77 (0.72–0.83)

Abbreviations: BMI, body mass index; CCI, Charlson comorbidity index; CI, confidence interval; HR, hazard ratio.

## Supplementary Materials

The following supporting information can be downloaded at: https://www.mdpi.com/article/10.3390/ijerph19063491/s1, Figure S1: Flow chart of study population; Table S1. Hazard ratios of long-term exposure to normalized difference vegetation index and on Parkinson’s disease in time-dependent Cox proportional hazards model and Cox proportional hazards model using various exposure windows; Table S2. Hazard ratios of long-term exposure to normalized difference vegetation index and on Parkinson’s disease in conventional Cox proportional hazards model using various exposure windows; Table S3. The association between 0.1 increase of normalized difference vegetation index and incidence of Parkinson’s disease among non-movers; Table S4. Hazard ratios of long-term exposure to normalized difference vegetation index and on Parkinson’s disease according to adjustment of different air pollutants
