# Supplementary material for: Association between Residential Greenness and Incidence of Parkinson’s Disease: A Population-Based Cohort Study in South Korea
Source: Int J Environ Res Public Health. 2022 Mar 15;19(6):3491. doi: 10.3390/ijerph19063491 (PMC8951185; doi:10.3390/ijerph19063491)
Supplement: Supplementary file 1 [file ijerph-19-03491-s001.zip › ijerph-1618803-supplementary.pdf]

Supplementary material for

Association between residential greenness and incidence of Parkinson's disease:

A population-based cohort study in South Korea

This file includes:

- Figure S1. Flow chart of study population.
- Table S1. Hazard ratios of long-term exposure to normalized difference vegetation index and on Parkinson's disease in time-dependent Cox proportional hazards model and Cox proportional hazards model using various exposure windows.
- Table S2. Hazard ratios of long-term exposure to normalized difference vegetation index and on Parkinson's disease in conventional Cox proportional hazards model using various exposure windows.
- Table S3. The association between 0.1 increase of normalized difference vegetation index and incidence of Parkinson's disease among non-movers.
- Table S4. Hazard ratios of long-term exposure to normalized difference vegetation index and on Parkinson's disease according to adjustment of different air pollutants.

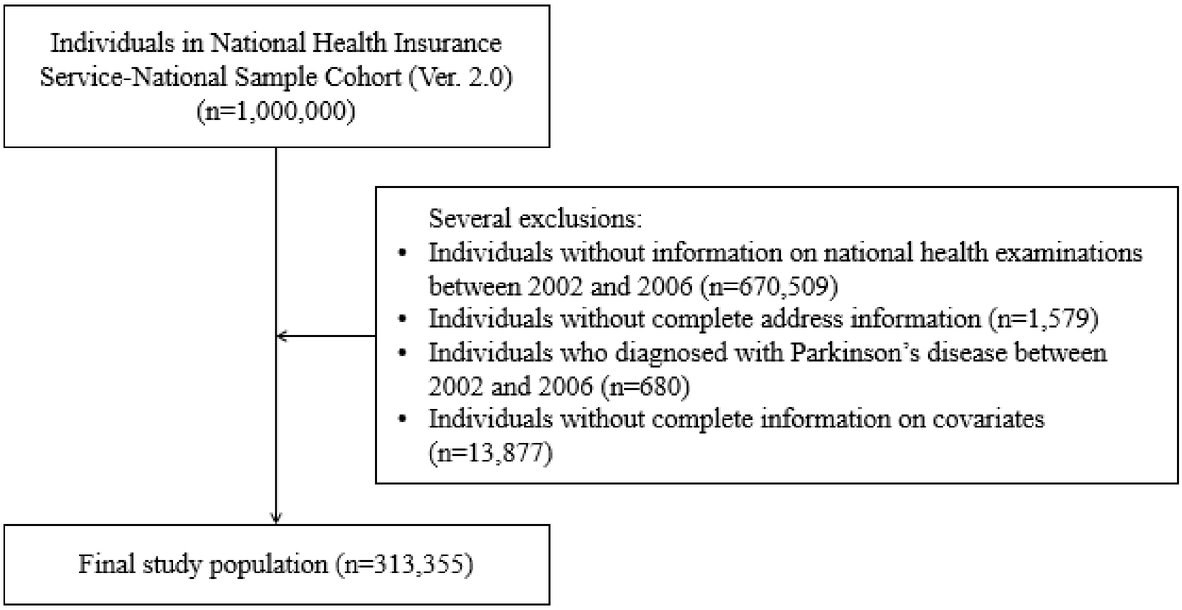

Figure S1. Flow chart of study population.

Table S1. Hazard ratios of long-term exposure to normalized difference vegetation index and on Parkinson's disease in time-dependent Cox proportional hazards model and Cox proportional hazards model using various exposure windows.

Exposure Period	HR (95% CI)
1 year before baseline	0.79 (0.74-0.84)
2 years before baseline	0.79 (0.74-0.84)
3 years before baseline	0.79 (0.74-0.84)
4 years before baseline	0.79 (0.74-0.84)
5 years before baseline	0.79 (0.74-0.84)

Table S2. Hazard ratios of long-term exposure to normalized difference vegetation index and on Parkinson's disease in conventional Cox proportional hazards model using various exposure windows.

Exposure Period	HR (95% CI)
1 year before baseline	0.92 (0.86-0.99)
2 years before baseline	0.93 (0.87-0.99)
3 years before baseline	0.93 (0.88-1.00)
4 years before baseline	0.93 (0.86-1.00)
5 years before baseline	0.93 (0.87-0.99)

Table S3. The association between 0.1 increase of normalized difference vegetation index and incidence of Parkinson's disease among non-movers.

		Person-Years	Cases	HR (95% CI)
Quantile				
	Q1: 0.16-0.34	328,018	252	1 [Reference]
	Q2: 0.34-0.41	363,002	327	0.90 (0.74-1.09)
	Q3: 0.41-0.50	377,095	465	0.73 (0.59-0.91)
	Q4: 0.50-0.64	440,852	650	0.59 (0.46-0.77)
	P for trend			0.01
Liner		1,508,967	1,694	0.77 (0.71-0.83)

Table S4. Hazard ratios of long-term exposure to normalized difference vegetation index and on Parkinson's disease according to adjustment of different air pollutants.

	PM_{10} (μg/m^3)	SO_2 (ppb)	NO_2 (ppb)	CO (0.1 ppm)
Quantile				
Q1: 0.16-0.34	1 [Reference]	1 [Reference]	1 [Reference]	1 [Reference]
Q2: 0.34-0.41	0.96 (0.83-1.11)	0.91 (0.79-1.06)	0.85 (0.73-0.99)	0.94 (0.81-1.09)
Q3: 0.41-0.50	0.77 (0.65-0.91)	0.71 (0.60-0.84)	0.63 (0.53-0.75)	0.70 (0.59-0.83)
Q4: 0.50-0.64	0.64 (0.52-0.77)	0.52 (0.43-0.63)	0.47 (0.39-0.57)	0.53 (0.44-0.63)
P for trend	0.01	0.01	0.01	0.01
Linear	0.79 (0.74-0.84)	0.74 (0.69-0.78)	0.71 (0.67-0.76)	0.74 (0.70-0.79)
